# Latinas in medicine: evaluating and understanding the experience of Latinas in medical education: a cross sectional survey

**DOI:** 10.1186/s12909-023-04982-y

**Published:** 2024-01-03

**Authors:** Gabriella Geiger, Lauren Kiel, Miki Horiguchi, Celia Martinez-Aceves, Kelly Meza, Briana Christophers, Priscilla Orellana, Maria Mora Pinzon, Sam J. Lubner, Narjust Florez

**Affiliations:** 1 University of Wisconsin School of Medicine and Public Health, Madison, WI USA; 2 Lowe Center for Thoracic Oncology, Dana-Farber Cancer Institute, Boston, MA USA; 3 Harvard Medical School, Boston, MA USA; 4 Yale University, New Haven, CT USA; 5 Weill Cornell/Rockefeller/Memorial Sloan Kettering Tri-Institutional MD-PhD Program, New York City, NY USA; 6 St. George’s University School of Medicine, St. George, Grenada; 7 Division of Geriatrics and Gerontology, University of Wisconsin School of Medicine and Public Health, Madison, WI USA; 8 Division of Hematology, Medical Oncology and Palliative Care, School of Medicine and Public Health, University of Wisconsin, Madison, WI USA; 9 Lowe Center for Thoracic Oncology, Harvard Medical School, Dana-Farber Cancer Institute, 450 Brookline Ave - DA1230, 02215 Boston, MA USA

**Keywords:** Latinas, Medical education, Discrimination, Burnout

## Abstract

**Background:**

The percentage of physicians identifying as Latina has not improved despite improvements in recruitment of Latina medical students, suggesting barriers to retention and career advancement. Discriminatory experiences and mental health inflictions throughout training may contribute to difficulties in recruitment, retainment, and advancement of Hispanic/Latinx trainees, a notably understudied population.

**Methods:**

An anonymous, online survey was distributed to Latinas in the continental U.S. between June 22 to August 12, 2022. Eligibility criteria included: self-identifying as Hispanic/Latina, female/woman, and completing or have completed medical school, residency, or fellowship in the continental U.S. in the past 10 years. Recruitment was done via the Twitter account @LatinasInMed and outreach to Latino Medical Student Association chapters. Descriptive statistics summarized the self-reported experiences.

**Results:**

The survey included 230 Hispanic/Latinx women, mostly medical students (46.9%). A majority (54.5%) reported negative ethnicity-based interactions from patients and/or patients’ families; 71.8%, from others in the medical field. High rates of depression (76.2%) and anxiety (92.6%) during training were reported by Latinas, especially medical students. Feelings of imposter syndrome and burnout were high at 90.7% and 87.4%, respectively.

**Conclusions:**

This is the first study evaluating the unique experiences of Latinas in medicine, who reported discrimination and mental health struggles, specifically during medical school, at alarmingly high rates. Our findings could aid in creating the needed interventions to support Latinas in medical training to reduce the existing exodus of Latinas from medicine.

**Supplementary Information:**

The online version contains supplementary material available at 10.1186/s12909-023-04982-y.

## Background

Efforts to improve diversity within academic medicine have been made, yet adequate representation of groups underrepresented in medicine (URM; self-identifying American Indian/Alaska Native, Black, Hispanic/Latinx, Native American, and/or Pacific Islander), specifically Hispanic/Latina physicians, continues to lag in relation to corresponding U.S. demographic changes. While Latinos make up 18.5% of the U.S. population [1], Latinas make up less than 3% of all practicing physicians, 2% of medical school faculty, and 3% medical school matriculants in 2019 [2–4]. Increased efforts to recruit URM medical students have not adequately increased the proportion of Latina physicians, suggesting barriers to retention such as attrition, which has been widely documented among other URM trainees [5–7].

Recently, investigators have begun to explore discrimination, mental health turmoil, and burnout, and imposter syndrome among URM trainees and physicians [8–13]. Data examining the responses of AAMC Graduation Questionnaires found that 23.3% of URM medical students reported experiencing discrimination based on their race/ethnicity, and that female, URM, multiracial, and LGBTQ + medical students made up the largest portion of mistreatment reports in medical schools across the United States, with female URM medical students experiencing the highest prevalence of racial/ethnic discrimination [8]. URM trainees face unique challenges, such as discrimination, throughout training that may significantly impact their mental health, as studies have shown that African American and sexual minority medical trainees are at an increased risk of experiencing feelings of depression and anxiety [11–13]. As burnout is furthermore associated with leaving the healthcare profession, lower patient satisfaction, and impaired quality of care [14–16], and imposter syndrome is highly correlated with burnout and is a significant risk factor for depression [17], particularly among minority populations, these occurrences are undoubtedly important to address. Nevertheless, most of the literature focuses on these instances among other marginalized groups in medicine, leaving the Latina experience in medical education out of the conversation and causing a notable lack of data and understanding about this group. This is alarming and warrants further investigation as Hispanic/Latinx is one of the fastest growing racial/ethnic groups in the U.S.; research supports how racial concordance can be associated with greater patient satisfaction in clinical care in the U.S. (18–19) Therefore, it is vital to understand the experiences within medical education of this understudied population, particularly considering their vulnerable intersectionality and the importance of their role in medicine.

To our knowledge, this is the first study aimed to describe the Latinas in Medicine in experience during medical training with a focus on the discrimination they face, psychiatric difficulties, and burnout among Latinas in all stages of medical education, in order to evaluate our current medical education environment and ultimately improve the retention of this growing population.

## Methods

### Setting and participants

This cross-sectional survey was approved by the Institutional Review Board (IRB) approval at the University of Wisconsin-Madison and distributed to self-identifying Latinas in the U.S. The study was approved with a waiver of documentation of informed consent due to the minimal risk associated with survey completion. Eligibility criteria included individuals 18 years or older that self-identified as a Hispanic/Latina, were currently completing or have completed any portion of medical training in the continental U.S. in the past 10 years, and able to complete the online survey in English.

### Survey

We developed the survey according to feedback from a multidisciplinary panel of experts in racial inequality in academic medicine. The survey was developed and distributed using SurveyMonkey™ (SurveyMonkey, San Mateo, CA); responses were electronically stored and collected in a de-identified manner on here and no paper records were created. To increase validity, the developed survey was sent for IRB review and subsequently pilot tested by the study team. The survey link was thereafter distributed through email outreach to Latino Medical Student Association (LMSA) chapters at 22 medical schools nationwide. Study advertisements were also posted on the official #LatinasInMedicine Twitter account @LatinasInMed, a community of over 9,500 members founded in 2019 to provide a space for connection, academic opportunities, and cross-institutional mentorship among Latinas in medicine globally [20]. The survey link was not shared publicly; interested individuals needed to request it via a private message, where their account was screened to mitigate the possibility of fake accounts gaining access to the survey. The screening was done by our team member who runs the @LatinasInMed account and consisted of checking that the account was not anonymous, and that the user stated in their profile their professional role. To eliminate any potential bias, their appearance was not used to verify their ethnicity. A $5 Starbucks E-gift card was offered as an incentive for participation. The survey was open from June 22 to August 12, 2022.

The study team used a two-step verification process to ensure that survey responses were valid. At the end of the survey, participants were asked to provide their institutional email address or state their affiliation to receive the study incentive. Further, responses with unreasonable answer patterns were reviewed and the survey was disregarded if the entire team agreed. To ensure confidentiality, email addresses were not associated with survey responses.

The 65-question survey included both multiple choice and open-ended questions to investigate important aspects of experiences during medical training, including: (1) discrimination, (2) mental health, and (3) burnout and imposter syndrome. To minimize survey length and respondent fatigue, validated measures were not used for these constructs. Respondents were asked to identify their current role (medical student, resident, fellow, attending/practicing physician, other), and prompted to only answer questions that applied to their indicated training level. Within the survey, participants were provided with comprehensive definitions of terms applicable to the question at hand. Due to the sensitive nature of reflecting upon negative institutional experiences induced by the survey, participants could skip questions.

Specifically, 12 multiple choice questions regarding discrimination, 4 regarding mental health, 2 related to burnout, and 2 related to imposter syndrome, were asked. The complete survey is available as an additional file (See Additional File 1).

### Analysis

Descriptive statistics were used to summarize the distribution of participant demographic characteristics. The proportions of participants who reported experiences with discrimination, depression, anxiety, burnout, and imposter syndrome in the study cohort and the corresponding 95% asymptotic confidence interval (CI) were estimated. R version 4.2.2 (R Foundation for Statistical Computing, Vienna, Austria) was used. Missing data were excluded from the analysis, and percentages (and their corresponding proportions) reported reflect the number of respondents who had chosen to answer each specific question.

### IRB statement

The Minimal Risk Research IRB at the University of Wisconsin-Madison determined this study to be exempt on 5/16/2022, reference number 2022 − 0664.

## Results

The survey was completed by 230 Latinas. Of such, 46.9% (105/224) of respondents were medical students; 13.8% (31/224), residents; 8.9% (20/224), fellows; and 24.1% (54/224), practicing physicians. Participants attended medical schools distributed throughout 27 U.S. states, with most being in the Northeast U.S. Notably,29.3% (60/205) attended medical school outside of the constitutional U.S., including Puerto Rico (Table 1).

**Table 1:** Demographic characteristics

Characteristics	Values, n (%) or median [IQR] (n = 230)
Age, n (%) n = 225	
18–22	10 (4.4%)
23–27	89 (39.6%)
28–31	43 (19.1%)
32–36	48 (21.3%)
37–41	24 (10.7%)
42–46	6 (2.7%)
47–51	4 (1.8%)
52+	1 (0.4%)
Afro-Latina, n = 224	24 (10.7%)
LQBTQIA + member, n = 225	28 (12.4%)
Place of birth outside the US, n = 225	101 (44.9%)
Current role or position, n (%) n = 224	
Medical student	105 (46.9%)
Resident	31 (13.8%)
Fellow	20 (8.9%)
Attending or Practicing physician	54 (24.1%)
Other	14 (6.2%)
Place of medical school, n (%) n = 205	
Northeast	51 (24.9%)
Midwest	43 (21%)
South	29 (14.1%)
West	22 (10.7%)
Outside of the US	60 (29.3%)
Place of residency, n (%) n = 101	
Northeast	38 (37.6%)
Midwest	17 (16.8%)
South	25 (24.8%)
West	17 (16.8%)
Outside of the US	4 (4%)
Place of fellowship, n (%) n = 66	
Northeast	23 (34.8%)
Midwest	11 (16.7%)
South	14 (21.2%)
West	17 (25.8%)
Outside of the US	1 (1.5%)

Experiences of discrimination were highly prevalent throughout medical training with 54.5% (114/209; 95% CI 47.8 to 61.3) of respondents having had negative experiences with patients or patients’ family members that they believe stemmed directly from their ethnic identity. In addition, an overwhelming majority (72.8% 147/202; 95% CI: 66.6 to 78.9) had been discriminated against by others in the medical profession, particularly during medical school (63.3% 93/145; 95% CI: 55.5 to 71.1). Alarmingly, 80.3% (118/147; 95% CI: 73.8 to 86.7) of those ever discriminated against did not report the incidence to supervisors of their medical schools or residency programs (Table 2). Notably, many Latinas also acknowledged the role of intersectionality, the interconnected nature of social categorizations that create overlapping and interdependent systems of discrimination or disadvantage as a result of the intersection of their ethnicity and gender [21], as 56.5% (83/147; 95% CI: 48.4 to 64.5) of those who had faced discrimination believed it to be based on a combination of their race/ethnicity, gender, and age.

**Table 2:** Discrimination during medical training

Scenarios	Overall, n (%) (n = 230)	Setting			
		Medical School	Residency	Fellowship	Job
Negative experience with patients or patients’ family members, n = 209	114 (54.5%)				
Discrimination by others in medicine, n = 202	147 (72.8%)	93 (63.3%)	60 (40.8%)	34 (23.1%)	43 (29.3%)
Basis of the discrimination n = 147					
Race/Ethnicity	42 (28.9%)				
Gender	16 (11.0%)				
Age	1 (0.7%)				
Combination of above	83 (56.5%)				
Other	6 (4.1%)				
Discrimination event reported, n = 147	26 (17.7%)				
Being mistaken for another Latin colleague, n = 208	127 (61.1%)	80 (63%)	60 (47.2%)	29 (22.8%)	37 (29.1%)
Being mistaken for another role (not med student/doctor), n = 211	179 (84.8%)	131 (73.2%)	86 (48%)	43 (24%)	62 (34.6%)
Asked to translate for another provider, n = 210	184 (87.6%)				

Experiences with microaggressions were also highly pervasive among Latinas, with a majority (61.1%, 127/208; 96% CI: 54.4 to 67.7) reporting being mistaken for other Latina colleagues throughout medical training. Moreover, an astounding 84.8% (179/211; 95% CI: 80.0 to 89.7) reported instances where others assumed them to hold a different role in the medical setting, including nursing staff, janitorial services, and food services, which represent female or Latino stereotypes. These experiences occurred throughout all phases of medical training, but most commonly during medical school and residency. As occurrences of perceived discrimination are confounding, negatively affect both mental and physical health, produce significantly heightened stress responses, and relate to unhealthy behaviors [22], such incidents may have worsened Latinas’ pre-existing imposter syndrome.

Mental health disorders were disturbingly extensive among Latinas. Rates of depression and anxiety were alarmingly high among women, with over three quarters (76.2%, 154/202; 95% CI: 70.4 to 82.1) having experienced feelings of depression during medical training and an overwhelming 92.6% (188/203; 95% CI: 89.0-96.2) having experienced anxiety. Specifically, 82.5% (95% CI: 76.5 to 88.5) and 82.4% (95% CI: 77.0 to 87.9) experienced difficulties with depression and anxiety, respectively, during medical school; though still prevalent, rates declined in subsequent stages of medical training (i.e., residency, fellowship, etc.).

High rates of burnout were identified, with an overwhelming 87.4% (180/206; 95% CI: 82.8 to 91.9) of Latinas experiencing burnout at any point within their medical education, most commonly during medical school. Rates of imposter syndrome, too, were distressingly prevalent among Latinas, with an astounding 90.7% (185/204; 95% CI: 86.7 to 94.7) experiencing imposter syndrome at some point throughout their training (Table 3). Importantly, 77.8% (144/185; 95% CI: 71.9 to 83.8) of these individuals believed that imposter syndrome affected their performance throughout training.

**Table 3:** Psychological and mental health effects during medical training

Scenarios	Overall, n (%) (n = 230)	Setting			
		Medical School	Residency	Fellowship	Job
Depression, n = 202	154 (76.2%)	127 (82.5%)	55 (35.7%)	29 (18.8%)	26 (16.9%)
Anxiety, n = 203	188 (92.6%)	155 (82.4%)	75 (39.9%)	42 (22.3%)	36 (19.1%)
Burnout, n = 206	180 (87.4%)	112 (62.2%)	68 (37.8%)	35 (19.4%)	46 (25.6%)
Imposter syndrome, n = 204	185 (90.7%)				

Experiences of mentorship were evaluated in order to explore possible support available to Latinas as a protective factor for negative experiences. Though 64.9% (135/208; 95% CI: 58.4 to 71.4) of Latinas indicated currently having a mentor in the medical field, most participants (54.8%, 114/208; 95% CI: 48.0 to 61.6) believed that during training, mentorship was not adequate, compared to that of their peers. Yet again, medical school, for the majority, was the phase of training most difficult to find mentors (67.8%, 141/208; 94% CI: 61.4 to 74.1), followed by residency (29.8%, 62/208; 95% CI: 23.6 to 36.0).

## Discussion

This is the first and the largest study to comprehensively evaluate the experiences of Latinas in medical education in the U.S. An overwhelming majority of Latinas in our study experienced discrimination from patients, patients’ families, and others in the medical field, most often in the earliest phases of training. Most Latinas who faced discrimination from others in the medical field attributed it to an aspect of intersectionality of their race/ethnicity, gender, and age, emphasizing previous research that observed a positive correlation between number of marginalized identities and risk of mistreatment and discrimination [23]. Therefore, the previously reported 23.3% of URM medical students that faced racial discrimination from responses of AAMC Graduation Questionnaires [8] is appreciably lower than the 54.4% and 72.8% of Latinas in our study who faced discrimination from patients and/or patients’ families and others in the medical field, suggesting that Latina trainees may be more at risk for discriminatory interactions than the general URM population. As such, current findings of URM experiences should not be broadly applied to Latinas. Further, as the majority of our study’s participants indicated that they did not report the incident(s) of discrimination, the reported numbers may be less than their true value. Medical education institutions must take such findings into account and adopt a zero-tolerance policy of discrimination along with openly accessible and widely advertised methods of reporting mistreatment in an anonymous and safe way. Trust needs to be earned by institutions for URM trainees to feel confident reporting negative experiences particularly associated with discriminatory practices. In addition, institutions must strategically cultivate a culture based on respect, trust, inclusivity, and leadership development, and cultural humility, routinely communicating their intolerance for biased patient conduct before appointments [24] (See Additional File 2). In the medical context, cultural humility may be defined as an awareness of how people’s culture can impact their health behaviors and in turn using this awareness to cultivate sensitive approaches when communicating with patients [25]. If faced with discrimination from patients, clinicians should aim to cultivate a therapeutic alliance and the patient’s medical condition, decision-making capacity, options for responding, reasons for the request, and effect on the physician, should be accounted for when considering patient re-assignment [26].

The unique struggles with mental health that Latinas in medicine face has not been described until our study. A 2016 meta-analysis of medical students’ experiences across the U.S. and 42 other countries estimated that 27.2% of medical students experience depression and depressive symptoms [27]; another meta-analysis reported that 33.8% of medical students experience anxiety [28]. Thus, our sobering results are consistent with previously reported data of marginalized groups in medicine [11–13] and reveal how Latina medical students experience depression and anxiety at *alarmingly high rates* compared to the general medical student population. Additionally, our study showed that a staggering 87.4% (180/206) and 90.7% (185/204) of participants suffered from burnout and imposter syndrome, respectively, astonishingly higher than the range of 22–60% reported by a meta-analysis evaluating imposter syndrome in the general population of physicians and physicians in training [29]. While we did not stratify rates of depression among those participants that reported experiencing burnout and/or imposter syndrome during their training, Latinas reported high rates of all three, suggesting some correlation.

We recognize that despite the increased attention to recruiting more Latinas into medicine, medical schools and training programs have not changed adequately enough to support them. An increase in diversity without the adaptation of inclusionary and supportive practices will lead to continued bleak experiences throughout medical training in URM trainees, making it important for institutions to implement factors early in medical training that may function as potential coping mechanisms and be protective of burnout, such as peer-based social support groups and mentorship [11, 30–32]. Adequate mentorship has the potential to increase career satisfaction, but URM trainees are unfortunately less likely to have such mentorship [33]. Though most Latinas (64%; 135/208) in our study indicated currently having a mentor in the medical field, more than half (54.8%; 114/208) stated that such mentorship and/or sponsorship was inadequate and medical school was identified as being the most difficult time to find mentorship. Therefore, medical schools must implement various avenues of accessible, comprehensive, and satisfactory mentorship to aid in the betterment of the overall well-being and advancement of URM trainees, specifically Latinas. However, institutions should not expect that the few Latina faculty they have should mentor all Latinas in training as this would greatly increase their existing burden and greatly accelerate burnout.

In our study, 29.3% of respondents reported attending medical school outside of the continental U.S. (including Puerto Rico). Though we did not stratify responses based on international medical graduate (IMG) status, it is known that these individuals often face unique challenges amidst the existing struggles of being a Latina in medicine, including navigating a new culture, immigration/visa stress, outside perception of not receiving adequate training due to attending medical school outside of the U.S., and adjusting to the U.S. healthcare system [34]. Future research should examine the experiences in medical education of IMGs; extra attention and support in the meantime, particularly relating to immigration stress, culture shock and adaptation, is warranted.

Our study has limitations. Since no validated scales for psychosocial conditions were used, we were not able to categorize the severity of depression and anxiety. In addition, part of our recruitment strategy emphasized individuals with a social media presence (Twitter) and participation in LMSA which may have added unknown selection bias. While not a direct limitation, the study population comprised mostly medical students; future research should explore whether results are indeed generalizable to Latinas in other stages of medical training. Nevertheless, our results support the findings that URM trainees, specifically those with intersectional identities, face challenges throughout medical training that negatively impact their experiences.

## Conclusions

The recruitment and retention of Latinas in medicine remains crucial to the creation of a physician workforce that represents the ever-diversifying U.S. population it serves. We found that most Latinas throughout all stages of medical education experienced discrimination, depression and anxiety, burnout, imposter syndrome, and lack of adequate mentorship at alarming high rates that may lead to attrition, further perpetuating the difficulty in creating a diverse physician workforce. While medical schools and training programs continue to put an emphasis on the importance of increasing diversity, these institutions must reform their culture and practices to provide ample support to Latinas and other marginalized groups in medicine.

### Electronic supplementary material

Below is the link to the electronic supplementary material.

Supplementary Material 1

Supplementary Material 2

## Data Availability

The datasets used and/or analyzed during the current study are available from the corresponding author on reasonable request.

## Abbreviations

URM: Underrepresented in medicine
IRB: Institutional Review Board
LMSA: Latino Medical Student Association
CI: Confidence interval
IMG: International medical graduate
