# Supplementary material for: Latinas in medicine: evaluating and understanding the experience of Latinas in medical education: a cross sectional survey
Source: BMC Med Educ. 2024 Jan 3;24:4. doi: 10.1186/s12909-023-04982-y (PMC10765906; doi:10.1186/s12909-023-04982-y)
Supplement: Supplementary file 1 — Supplementary Material 1 [file 12909_2023_4982_MOESM1_ESM.docx]

**Additional File 1**

**Survey Administered to Latinas in Medicine in a Study Evaluating the Experiences of Latinas in Medical Education.**

1. Responses are anonymous and your participation is voluntary. You will be emailed a $5 Starbucks gift card for completing the survey. To ensure your participation is anonymous, your email address will not be linked to your survey responses. If you decide to participate in this research survey, you may withdraw at any time. You can skip questions you do not feel comfortable answering. If you choose not to participate in this study, you will not be penalized in any way. If you have any questions or concerns, please email the study coordinator Gabriella Geiger (ggeiger@wisc.edu). This survey will take approximately 15-20 minutes to complete. By answering this survey, you agree to the following:-I am older than 18 years of age;-I identify as female and/or woman-I identify as Hispanic/Latinx;-I have completed or am completing part of my medical education in the United States in the past 10 years. (medical school, residency, fellowship)-I voluntarily agree to participate in this study;-I can complete the survey in English;-I have read all the above information.
   1. Agree
   2. Disagree
2. Select your age
   1. 18-22
   2. 23-27
   3. 28-31
   4. 32-36
   5. 37-41
   6. 42-46
   7. 47-51
   8. 52+
3. Select your gender identity (select all that apply)
   1. Woman
   2. Man
   3. Non-binary/third-gender/agender
   4. Transgender
   5. Other
4. Do you identify as a member of the LGBTQIA+ community?
   1. Yes
   2. No
5. Do you self-identify as Afro-Latina (people from Latin American countries with African ancestry)?
   1. Yes
   2. No
   3. Prefer not to answer
6. Were you born in a different country?
   1. Yes
   2. No
7. What is your current professional role/position?
   1. Medical Student
   2. Resident
   3. Fellow
   4. Attending/Practicing Physician
   5. Other (Please Specify)
8. Medical School year in Fall of 2022
   1. MS1
   2. MS2
   3. MS3
   4. MS4
   5. MS5+
   6. Year off
   7. Other (please specify)
9. Year as a Resident:
   1. PGY1
   2. PGY2
   3. PGY3
   4. PGY4
   5. Year Out
   6. Other (please specify)
10. Year as a Fellow

[text box]

1. What State do/did you attend Medical school?
   1. Outside of the U.S.
   2. Alabama
   3. Alaska
   4. ETC.
2. What state do/did you complete your residency training (if applicable)?
   1. Outside of the U.S.
   2. Alabama
   3. Alaska
   4. ETC.
3. What state do/did you complete your fellowship training (if applicable)?
   1. Outside of the U.S.
   2. Alabama
   3. Alaska
   4. ETC.
4. If you are an attending already, what is your specialty (if applicable)?

[text box]

1. Are either of your parents in the medical field?
   1. Yes
   2. No
2. Are you the first in your family to obtain an undergraduate degree?
   1. Yes
   2. No
3. Are you the first in your family to attend or finish graduate school?
   1. Yes
   2. No
4. Did you (do you) experience financial hardships during: (select all that apply)
   1. None of the above
   2. Medical School
   3. Residency
   4. Fellowship
5. At any point in your training, have you felt that you have been at a financial disadvantage compared to your peers?
   1. Yes
   2. No
6. In what setting(s) (select all that apply)
   1. None of the above
   2. Medical School
   3. Residency Training
   4. Fellowship Training
   5. Job
7. Did/do you have financial support for academic resources such as board prep classes, questions banks, tutoring services, etc?
   1. Yes
   2. No

[text box for additional comments]

1. Please rate your overall experience during medical school
   1. Completely Dissatisfied
   2. Somewhat Dissatisfied
   3. Neutral
   4. Somewhat Satisfied
   5. Completely Satisfied
2. If you could describe your medical school experience in a few words, what would you say:

[text box]

1. Do you think that being Latina ever affects your ability to receive the grades that you deserved?
   1. Yes
   2. No

[text box for additional comments]

1. Have you had negative experiences with patients that you felt directly stem from your identity as a Latina
   1. Yes
   2. No
2. Have you ever been discriminated against by a patient or their family members due to your Latinx/Hispanic heritage?
   1. Yes
   2. No
3. Do you speak another language besides English?
   1. No
   2. Yes, drop down menu featuring top 12 most common languages in the world
   3. Other
4. Have you ever been asked to translate for another provider?
   1. Yes
   2. No
5. Have you ever been mistaken for another (Latinx/Hispanic) colleague? If yes, during which phase of training (select all that apply)?
   1. None of the above
   2. Medical School
   3. Residency Training
   4. Fellowship Training
   5. Job
6. Have you ever been assumed to have another role in a healthcare setting other than medical student/doctor? (ex: a member of janitorial staff, interpreter, nursing staff, etc)
   1. Yes
   2. No
7. In what setting(s) were you assumed to hold another role? (select all that apply)
   1. Medical School
   2. Residency Training
   3. Fellowship Training
   4. Job
   5. N/A

[text box for additional comments]

1. Would you say that you currently have a mentor in the medical field?
   1. Yes
   2. No
2. Do you think you have had adequate mentorship during your training compared to your peers?
   1. Yes
   2. No
3. In which phase(s) of training did you find it most difficult to find mentors? (select all that apply)
   1. Medical School
   2. Residency
   3. Fellowship
   4. Job
   5. N/A
4. Did/does your residency training program have a formal mentorship program?
   1. N/A
   2. Yes
   3. No
5. Did/do you find the mentorship program at your training program to be effective/helpful?
   1. N/A
   2. Yes
   3. No
6. How much research experience do you have?
   1. None
   2. A little bit
   3. A fair amount
   4. A great deal
7. During what stage of training did you start conducting research?
   1. Before medical school
   2. Medical school
   3. Residency
   4. Fellowship
   5. Job
   6. N/A
8. How many publications do you have?
   1. 0
   2. 1-3
   3. 4-5
   4. 6-7
   5. 8-9
   6. 10+
9. If you do not have any publications, why? (select all that apply)
   1. Not interested
   2. Lack of opportunities
   3. Lack of mentorship
   4. Did not know where to start
   5. Did not have time
   6. Struggled with funding
   7. Not enough training/guidance
   8. Other
   9. N/A
10. Are/were there Latinas in leadership roles at your institution during (select all that apply):
    1. None of the above
    2. Medical School
    3. Residency Training
    4. Fellowship Training
    5. Job
11. Have you or are you currently holding a leadership role? Include current/past roles
    1. Yes
    2. No
12. Have you had to extend your medical or residency training in the U.S?
    1. Yes
    2. No

[text box for additional comments]

1. Have you ever switched specialties?
   1. Yes
   2. No
   3. N/A

[text box for additional comments]

1. As a Latina in medicine, have you ever felt discriminated against by others in the medical field?
   1. Prefer not to answer
   2. Yes
   3. No
2. When did you feel discriminated against? (select all that apply)
   1. Medical School
   2. Residency
   3. Fellowship
   4. Job
3. Did you report this instance?
   1. Yes
   2. No
4. What was the discrimination based on?
   1. Race/Ethnicity
   2. Gender
   3. Age
   4. Both gender and race/ethnicity
   5. Other (please specify)
5. Do know any colleagues in the medical field who have been discriminated against?
   1. Yes
   2. No
6. What was the discrimination based on?
   1. Race/Ethnicity
   2. Gender
   3. Age
   4. Both gender and race/ethnicity
   5. Other (please specify)
7. Have you experienced feelings of depression during your medical training?
   1. Prefer not to answer
   2. Yes
   3. No
8. During what point of your training did you experience feelings of depression? (select all that apply)
   1. Medical school
   2. Residency
   3. Fellowship
   4. Job
9. Have you experienced feelings of anxiety during your medical training?
   1. Prefer not to answer
   2. Yes
   3. No
10. During what point of your training did you experience feelings of anxiety? (select all that apply)
    1. Medical school
    2. Residency
    3. Fellowship
    4. Job
11. Have you experienced feelings of burnout (a long-term stress reaction marked by emotional exhaustion, depersonalization, and a lack of sense of personal accomplishment) during your medical training?
    1. Yes
    2. No
12. During what point in your training did you experience feelings of burnout? (select all that apply)
    1. Medical School
    2. Residency
    3. Fellowship
    4. Job
13. If you wish to elaborate, tell us more about your experience with feelings of depression, anxiety, and/or physician burnout

[text box]

1. Did/do you feel that your residency program had/has a hostile work environment?
   1. Yes
   2. No
   3. N/A
2. At any point in your training, did you feel threatened by your program director or faculty in your program?
   1. Yes
   2. No
   3. N/A
3. Did you seek help?
   1. Yes
   2. No
4. From whom did you request help from (select all that apply)
   1. N/A
   2. Ombudsman Office
   3. ACGME Officer
   4. Faculty
   5. Chairman
   6. Wellness Officer
   7. Assistant Program Director
   8. Program Director
   9. Legal Experts
   10. Other
5. Do you think that your ethnic background increased the pressure of having children before or during medical training?
   1. Yes
   2. No
6. Have you experienced “imposter syndrome” (a phenomenon where an individual doubts their accomplishments or talents and has a persistent internalized fear of being exposed as fraud)?
   1. Yes
   2. No
7. Do you feel that imposter syndrome affected your performance in medical training?
   1. Yes
   2. No
   3. N/A
8. Feel free to share any additional information about your experience as a Latina with medical education, here:

[text box]

1. Please enter your email address so we can send you the gift card: *note that your email will not be attached to your responses. All of your responses will remain anonymous. Email will only be used for sending the gift card. If you are not using your institutional email, please enter the email of your choice and the name of your institution. We won’t link this to your responses, but we do it to avoid bots and spam. Thank you!
