# Supplementary figures and images for: Latinas in medicine: evaluating and understanding the experience of Latinas in medical education: a cross sectional survey
Source: BMC Med Educ. 2024 Jan 3;24:4. doi: 10.1186/s12909-023-04982-y (PMC10765906; doi:10.1186/s12909-023-04982-y)

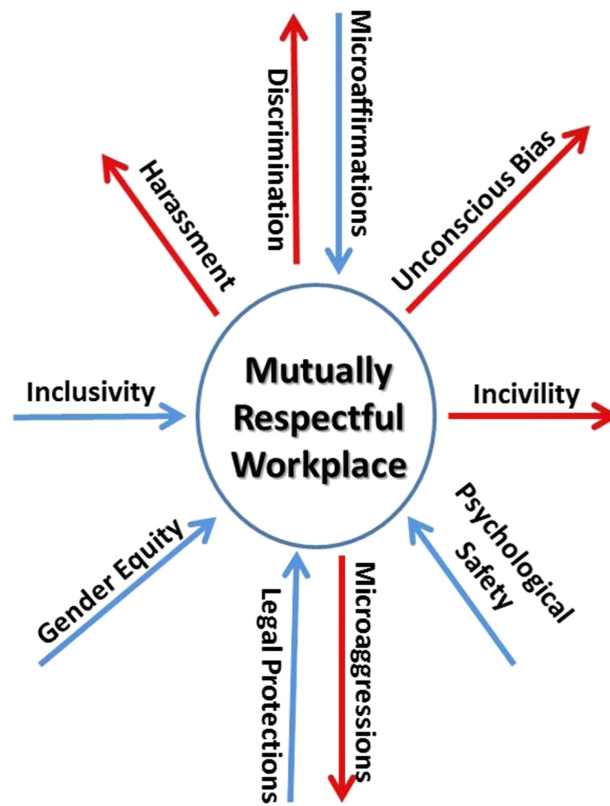

FIGURE 1. Schema of Factors Interacting in the Creation of a Mutually Inclusive Workplace

Supplement: Supplementary file 2 — Supplementary Material 2 [file 12909_2023_4982_MOESM2_ESM.pdf]
